# Tackling segmentation to advance universal health coverage: analysis of policy architectures of health care in Chile and Uruguay

**DOI:** 10.1186/s12939-020-01176-6

**Published:** 2020-07-31

**Authors:** Pamela Bernales-Baksai

**Affiliations:** 1 Department of Social & Policy Sciences, University of Bath, Bath, UK; 2 Programa de Trabajo, Empleo, Equidad y Salud, Facultad Latinoamericana de Ciencias Sociales Sede Chile (FLACSO Chile), Santiago, Chile

**Keywords:** Universal health coverage, Universalism, Segmentation, Policy architecture, Policy outputs, Chile, Uruguay

## Abstract

**Background:**

With the turn of the century, most countries in Latin America witnessed an increased concern with universalism and redistribution. In the health sector, this translated into a wide range of reforms to advance Universal Health Coverage (UHC) that, however, have had to cope with health systems that stratified the population since their foundation and the further segmentation inherited by market-oriented policies in the 1980s and 1990s.

Studies on social welfare stress the relevance of cross-class alliances between the middle and working classes to reach universal and sustainable social benefits. Consequently, the endurance of separate health schemes across groups of the population in most countries in Latin America may seriously hamper the efforts towards UHC.

**Aim:**

This article addresses the potential of current policy architectures of health care to tackle segmentation between social classes in access to health services in two of the best performers of health coverage in the region, namely Chile and Uruguay.

**Methods:**

The article is a comparative case study based on a literature review and applies an analytical framework that links universal outputs to the policy architectures of health care. The study assesses universal outputs in terms of coverage, generosity and financial protection, identifying equity gaps in each of these dimensions across groups of the population.

**Findings:**

Latest processes of reform for UHC in Chile and Uruguay perform highly regarding population coverage. Nevertheless, equity gaps in access to quality services and financial protection remain. In both countries, such gaps relate to the eligibility criteria. In Chile, segmentation is reinforced by the persistence of separated pools of resources that hinder solidarity. Besides, the significant role of private actors and differences in quality between public and private service providers continue to push middle and upper-middle classes to private options. Uruguay’s health reform reinforced the public system and promoted financial solidarity by pooling and progressively allocating resources. Despite this, fragmentation in service provision continues the segmentation of access to health care.

**Conclusions:**

The study shows differences in the options of reforms for UHC in Chile and Uruguay and the relevance of policy architectures to reverse, or conversely deepen, segmentation across groups of the population.

## Background

With the turn of the century, most countries in Latin America (LA) witnessed an increased concern with universalism and redistribution [1, 2]. In the health sector, this translated into a wide range of reforms aimed at enhancing access to health care and including groups of the population that historically remained behind [3–5]. The 2012 United Nations (UN) General Assembly Resolution to advance Universal Health Coverage (UHC) and the inclusion of UHC as one of the targets in the Post-2015 SDGs reinforced even further the high-level political commitment which contributed to the pursuit of country-level policies to improve people’s access to health care. Thus, the quest for UHC has extraordinarily thrived over the past decade [6].

The UN Resolution made a case for a comprehensive definition of UHC that considers access to the entire population to promotive, preventive, curative and rehabilitative health services and quality and affordable medicines. It also recognised the need to address the social, environmental and economic determinants of health [7]. Recently, the UN Political Declaration of the High-level Meeting on UHC 2019, reaffirmed the member states´ compromise with this initiative and declared that UHC implies that all people have access to the necessary health services, without discrimination, and with a special emphasis on the poor, vulnerable, and marginalised segments of the population [8]. Nonetheless, so far, the translation of UHC into implementation has been led by multiple approaches and hindered by the lack of a consistent framework to support policy-making [9].

In LA, the lack of a single approach has been expressed in different paths of reform across countries, ranging from the creation of unified health systems with equal benefits for all citizens to explicit entitlements to specific health services delivered through separated schemes [3]. Thus, although the recommendations set by the international agencies are aimed at a comprehensive understanding of the UHC that entails equity in access, quality health services, and financial protection as essential related objectives [10], these have not always been the guiding principles of health reforms and more discussion seems to be required in order to unpack the operational translation of UHC into concrete policies.

In such a landscape, the discussion on universalism coming from social policy and welfare literature may be a valuable contribution. Although traditionally most of the welfare studies did not address health care specifically, over recent years scholars have increasingly incorporated this policy area into cross-national analyses, examining issues such as decommodification in health care [11, 12], the evolution of health care in countries with different welfare models [13], the influence of policy legacies on the development of universalist policies of health care [14], the links between the policy architectures and universal outputs [5], segmentation in health care [15], and the moved of Social Health Insurance systems towards UHC [16], among others. Likewise, some health experts have made significant contributions in relating health policy to the broader social and political context (see for instance) [17–20]. The dialogue between social sciences, and in particular social policy literature, and health systems research may still become far more prolific.

In the social policy arena, there has been long-term discussion and multiple approaches to addressing universalism. Traditionally, the term has been associated with social programmes funded by general revenues that provide flat benefits to all residents of a country as a right [21]. However, recently the term has been used more vaguely to refer to programmes aimed at reaching the entire population, independently whether benefits are sufficient and equal for everyone or not [5]. In other words, these approaches do not account for the extent, quality and equity of the services or benefits guaranteed, which become critical in most unequal countries, like many in LA, that carry a long trajectory of exclusion of some sectors of the population from their social policy regimes [22–25].

Against such limitations, over the past years, scholars have been increasingly concerned with defining universalism more accurately. In this process, the term has become associated with others such as equity, generosity, decommodification, and segmentation, among others. Besides, some scholars have argued that the necessary configuration of policy instruments to produce universal policy outputs may be more than a single one (see [1, 5, 14]).

Concentrating on the study of developed countries, Korpi and Palme [26] made an outstanding contribution to raising the debate by stressing the complex relationship between universalism and reduction of inequality. The authors demonstrated that universalistic models where all citizens are included in the same programmes, rather than the addition of multiple schemes to reach the whole population, have greater redistributive capacity. Thus, policies aimed at universal outputs and inequality reduction would need to bring low-income groups and better-off citizens into the same programmes and institutional structures. Huber and Stephens [1] support this conclusion in a study that examines the redistributive capacity of social policies across countries in LA.

Béland et al. [13] also discuss the relevance of keeping the entire population, or at least the majority, within the same scheme. For the authors, it is more appropriate to consider universalism in terms of degrees and distinguishing between universality in entitlements and universality in use. They highlight that when a significant share of the population relies on private options rather than on services publicly provided, it expresses a lower degree of universalism in public programmes even if those programmes provide formal entitlements to everyone.

In LA, Cecchini et al. [27] and Filgueira [28] identify three main approaches regarding universalism, namely the Social Protection Floor (SPF) led by the International Labour Organization that promotes universal basic income and essential social services complemented by contributory and/or voluntary schemes of insurance; the Basic Universalism (BA), that advocates for basic universal social benefits financed by general revenues but, unlike the SPF, aims at gradually increasing the generosity of universal benefits rather than relying on contributory complements [29]; and the Efficient Universalism (EU) which proposes social benefits financed by taxes on consumption for all workers (i.e. either formal or informal). Nevertheless, this latter approach could hardly be considered genuinely universal, as instead of addressing the whole population, it relates benefits to participation in the labour market. In turn, despite SPF and BU meeting the criterion of covering everyone, their emphasis on basic services risks neglecting the quality of those services and may end up generating multi-tiered systems for different segments of the population [5].

Drawing upon the examination of the health and old-age pension policies in the South, in recent years Martínez Franzoni and Sánchez-Ancochea [5] have proposed a definition of universalism based on three dimensions: massive coverage of the population, sufficient generosity of benefits and services to deliver adequate levels of social welfare, and equity of benefits and services across the groups of the population. Thus, by explicitly adding the dimensions of generosity and equity, this conceptualisation refutes the universal character of programmes that do not provide sufficient benefits or quality services to meet the welfare needs of the entire population and that end up creating inequities based on the resources of different social groups.

In a subsequent study, these authors stated universalism and segmentation as the two ends of a continuum [15]. Accordingly, universal policy outputs, on the one hand, comprise the coverage of the entire population with generous benefits or services and without the necessity of relying on the market [5]. Segmentation, on the other, takes place when coverage, benefits or services are uneven across social groups or when a significant share of people are exposed to the market forces to access social services or benefits that are essential [15]. In addition, Martínez Franzoni and Sánchez-Ancochea [5] warn about the usual conflation of universalism with the policy architecture, namely the instruments that the policy uses to produce the outputs (e.g. services defined as a social right vs targeting, programmes funded by general revenues or by payroll taxes). Accordingly, the policy instruments can constrain or enhance universal/segmented policy outputs but do not constitute universalism by themselves. It is the conceptualisation of universalism/segmentation that this article adopts hereinafter since it allows a more nuanced discernment of the policy outputs (i.e. drawing attention to each dimension) and allows for the relation of such outputs to the policy design.

Regarding the conditions for universal policies to thrive, namely the conditions that push for the political willingness that allow the setting of the appropriate policy architecture according to the specific context, seminal studies on social welfare stress the relevance of cross-class alliances and especially of the middle classes. Esping-Andersen [30] notices that demands for more generous social benefits require the formation of alliances between the working and the middle class and the electoral support of the latter. Korpi and Palme [26], in the so-called ´Paradox of redistribution` postulated that even though it is expected that policies that target the poor have a higher redistributive effect, in practice the result is the opposite. The explanation for this phenomenon is that countries that rely more markedly on targeting also spend less on social policies, and therefore attain less redistribution, because of the limited political support of programmes aimed at a small part of the population vis-à-vis the middle classes´ willingness to pay higher taxes for benefits that reach the whole population [1]. From another theoretical standpoint, Baldwin [31] also unveils that in most advanced welfare states the middle class was a fundamental actor for institutionalising universal policies and benefits rather than assistance programmes for the neediest.

In a scenario of less-developed, or even exclusionary social policies, Barrientos [24] and Filgueira [23, 32, 33] shed light on how the marketisation of social welfare in LA countries has reinforced the production of two-tiered systems that supply uneven benefits and services to different groups of the population. Filgueira [32] and Ferreira et al. [34] present a clear analysis drawing attention to the creation of perverse cycles that end up worsening the quality of public services and consolidating the segmentation of social welfare. In the so-called ´Trap of public goods`, Filgueira [32] explains that the lasting lack of quality of public services has spurred the upper and middle classes towards private options; removing demanding and influential users from the public services, and creating a feedback loop for the poor quality of public services.

Specifically addressing the policy outputs in health care, Martínez Franzoni and Sánchez-Ancochea [15] empirically demonstrated that segmentation remains widespread in LA, although with significant cross-country variability. These findings concur with the conclusions reached by the health systems scholarship that stress that most systems in the region continue to be fragmented and to segment the population, supplying uneven quality and usually leading to financial hardship at the point of service [4, 6].

Building on these findings, it becomes evident that segmentation and the role of middle classes are critical considerations for advancing UHC in LA. In fact, the processes of reform developed throughout the 20-first century have had to cope with health systems that stratified the population since their foundation [35] and the further segmentation inherited by market-oriented policies that deteriorated the quality of public services in the 1980s and 1990s [1] and pushed millions of people out of the state-funded services [36]. In this landscape, this article addresses the potential of current policy architectures of health care to tackle segmentation in access to health services across social classes in Chile and Uruguay.

The main reason for selecting these two countries is that they share some pivotal features, namely they are among the best performers of health coverage and health outcomes and the best-developed social policy regimes in the region, whereas they notoriously differ in their path of reform for UHC and the resulting policy architecture.

The study is an empirical contribution that fills the gap in the literature by presenting a comparative analysis that scrutinises the policy architectures of health care and their strengths and weaknesses in order to advance UHC from the specific perspective of how they deal with the segmentation of health care across social classes. In doing so, it applies the social science lens to unveil institutional barriers behind the inequities in accessing health services.

The remainder of this article is divided into four sections: Section 2 explains the methods utilised for conducting the study. Section 3 presents the policy architecture and the outputs of the contemporary policy of health care in Chile and Uruguay. Section 4 analyses the links between the policy architecture and outputs and the implications for overcoming segmentation of social classes and advancing UHC. Finally, section 5 offers some concluding remarks.

## Methods

The article examines the contemporary architectures and policy outputs of health care in Chile and Uruguay, focusing on identifying either the contributions or institutional constraints that the instruments that make up the policy architecture create to advance UHC. In particular, how the policy architecture creates conditions that foster universalism or segmentation across the population.

The enquiry applies a comparative case study methodological design based on a literature review. As discussed in section 1, the study is grounded in the analytical framework developed by Martínez Franzoni and Sánchez-Ancochea [5] that links universal outputs to policy architectures. Policy architectures are defined as the combination of policy instruments that establish the beneficiaries, benefits and services, and mechanisms to deliver such benefits and services. These policy instruments are eligibility criteria: under what criteria people are entitled to the benefit/service; funding arrangements: sources of revenues, co-payments, pooling of resources and possibilities of solidarity in resource allocation; benefits: who decides and what benefits/services are included; delivery: fragmentation/unification in service supply, who delivers services for whom, the state’s purchasing of private services; and outside options: size, regulation, and level of integration of private options in the health system.

Regarding policy outputs, the article assesses universal/segmented outputs relying on the three dimensions suggested by Martínez Franzoni and Sánchez-Ancochea [5], namely coverage (i.e. population entitled to receive the services), generosity (i.e. comprehensiveness and quality of the services), and equity (i.e. evenness of the services received by different social groups). Moreover, this study adds financial protection as a fourth dimension because of its significance for advancing UHC according to the World Health Organization and UN’s framework.

The dimension of coverage encompasses indicators of formal coverage and sociodemographic profile of the included population. The dimension generosity considers the availability of resources per capita as a proxy measure for the quality and comprehensiveness of the services actually delivered. The dimension of financial protection considers the existence of state subsidies, level of OOP spending, and service utilisation. Finally, concerning equity, this dimension is not assessed through independent indicators but by differentiating the outputs and identifying the equity gaps in each of the other three dimensions across groups of the population participating in the different schemes, type of insurance or providers that co-exist in each country.

## Results

### Policy architecture and outputs of health care

The current health care arrangements in Chile are, to a significant extent, the result of the reforms introduced by the military dictatorship in the early 1980s. These reforms replaced the historical public system inspired by a Bismarckian model by a dual public-private system composed of a public insurance in the hands of the National Health Fund (Fondo Nacional de Salud, FONASA-Chile) and private insurance managed by private for-profit institutions (Instituciones de Salud Previsional, ISAPREs) [37–39].

In the 1990s, after the return of democracy, the country strengthened the institutionality to regulate the so far highly unregulated private insurance. Also, the democratic governments substantially increased public spending on health to recover the underfunded public sector [38, 40]. Nevertheless, governments avoided structural reforms and the system kept the dual organisation introduced in the 1980s [24, 39]. In 2004, the introduction of the Universal Access Plan of Explicit Guarantees in Health (Plan de Acceso Universal a Garantías Explícitas en Salud, AUGE-GES) marked the beginning of the first post-retrenchment reform inspired by universalistic principles. The AUGE-GES Plan guarantees to all citizens quality and standardised health services and financial protection for a group of priority health problems [37]. Initially, the number of covered health problems was 25 [41] and currently reaches 85 [42].

The aim of this reform was not to extend the coverage that already surpassed 94% [43], nor to lead a structural transformation to the dual organisation of insurance and service delivery, but to enhance equity in access through the AUGE-GES Plan (Law 19.966), and to strengthen the regulation of the ISAPREs (Laws 19.895 and 20.015), along with better differentiation of some functions regarding the stewardship of the system (Law 19.937) [44–47].

In Uruguay, until the year 2007, most health care was organised around two detached systems: a general revenues-based public one (Administración de los Servicios de Salud del Estado, ASSE) for the poor and informal workers, and a private non-for-profit sector (Instituciones de Asistencia Médica Colectiva, IAMCs) with fragmented occupation-based schemes for workers in the formal economy and middle classes in general [48]. After a steep economic downturn in the early 2000s that worsened access to quality health care all across the population [49] and put the IAMCs into a crisis of sustainability, in 2005 the new left-wing government recognised health as a ´legal right and a public good` and, by 2007, started the most significant transformation to the policy of health care ever in the country [50].

The reform set the Comprehensive National Health System (Sistema Nacional Integrado de Salud, SNIS) (Laws 17.930 and 18.211) and changed the model of financing (Law 18.131) [51–53]. It allowed more progressivity in revenue collection and allocation by pooling resources from payroll contributions and general revenues into a single National Health Fund^1^ (Fondo Nacional de Salud, FONASA-Uruguay^2^) that includes all people of the contributory scheme (i.e. formal workers, dependent family members, and pensioners). Also, the fiscal investment in the public sector rose, allowing more availability of resources. Thereby, the reform pushed for greater equality in the benefits and services guaranteed across the suppliers [49, 54–57].

Table 1 presents a picture of the policy instruments that shape the contemporary policy architecture of health care in these two countries. In turn, Table 2 shows the policy outputs but, unlike other analyses that make cross-country comparisons using the overall outputs, this table also focuses on distinguishing, within each country, differences across type of insurance/schemes and groups of the population when they exist.

## Discussion

### Policy architecture and implications for universal (and equitable) health care

The previous section (Tables 1 and 2) shows that some of the policy instruments that make up the current policy architecture in Chile and Uruguay contribute to reaching universal outputs, whereas others set institutional conditions that hinder the advances towards UHC or even promote the segmented access according to the groups´ positions within the society, either because of their economic resources, occupation or status of employment.

In Chile, the policy architecture considers payroll contributions (i.e. social security) as the primary eligibility criterion. This enables formal coverage either at the public insurance (FONASA-Chile) or private insurance (ISAPREs). Nonetheless, each insurance keeps independence to define alternative or additional criteria. Formally everyone who participates in social security via contributions can enrol either in FONASA-Chile or ISAPREs. Nevertheless, by requesting extra premia, ISAPREs cream the population by health-risks and income level [38, 58, 59]. FONASA-Chile, on the contrary, introduces need as an alternative criterion for coverage. Such differences in the eligibility criteria translate into markedly uneven socio-demographic profiles of the insured population (Table 2), at the expense of FONASA-Chile, which concentrates on the groups with higher health-risk and lower-income [43]. Besides, as health coverage is not guaranteed as a right, some groups are excluded by not meeting any criteria (i.e. those who are not formally employed nor poor and do not have enough economic resources to invest in private insurance). Although, the sustained extension of the criterion of need by FONASA-Chile prevents this from being an extended situation.

Differently, in Uruguay, no one is formally excluded from coverage, as the reform complemented contributions to social security with citizenship as criteria for eligibility [56, 60]. The endurance of the distinction between contributory and subsidised population, however, implies that not everybody has similar chances of coverage, namely only those contributing to social security can enrol at the IAMCs or for-profit private insurance. Besides, as in Chile, by requesting extra premia, for-profit private institutions can cream the population according to their ability to pay and health-related risks.

Data demonstrates that coverage at the IAMCs currently is more evenly distributed across different income groups than before the 2007 reform, as coverage in the 1st income quintile passed from 6.3 to 21.4% between 2006 and 2011. Still, higher-income groups remain overrepresented (10.5% of people in the 1st income-quintile vs 23.6% in the 5th income-quintile) [56]. On the other hand, the population at ASSE continue to be mostly entitled via subsidies (67.8% in 2014) [61] and the majority of FONASA-Uruguay holders opt to enrol the IAMCs (80% at IAMCs vis-à-vis 17.6% at ASSE, and 2.4% at private insurances in 2014) [56]. Consistently, a study conducted in urban areas shows a positive relation between coverage by IAMCs and private insurance and higher levels of welfare^3^ [62]. Regarding risk profiles, differences by sex and age of people are not marked [63] although males and working-age population are slightly more represented at the IAMCs than females, younger, and older people [64].

Differences in eligibility rely on funding mechanisms. In Chile, financing arrangements enable people to ´opt-out` and make payroll contributions to private insurance. Furthermore, ISAPREs can request extra premia and keep their resources in independent funds separated from each other and the public fund managed by FONASA-Chile. Thus, although the system encompasses solidarity within the public insurance that pools the resources of all its holders (i.e. via contributions and subsidies), the broader financing structure imposes institutional restrictions for redistribution. These arrangements lead to less availability of resources in FONASA-Chile vis-à-vis private options that, with around 15% of the population, collect 46% of resources [38] and have between 30% (excluding Out of Pocket spending (OOP) and 39% (including OOP) more resources per capita than the public insurance, not even considering the differences in risk profiles [58].

Eligibility criteria and financing mechanisms also translate into a lower ratio of medical hours per enrolled population and long waiting lists in the public sector, leading to inequities in the generosity of services. Private providers hold more than half of physicians and medical hours [38, 65] meanwhile in the public sector waiting lists surpass 1.6 million people and about 40% wait for more than a year [58].

Otherwise, in Uruguay financing arrangements have been at the centre of the health reform. The pooling of all payroll contributions into a single fund (FONASA-Uruguay) created the institutional conditions for a higher mix of people with different backgrounds within the IAMCs. In turn, FONASA-Uruguay allocates resources by calculation of health risk instead of by the amount of contribution negotiated by different occupational categories with the IAMCs, as it was before the reform [55, 66]. Consequently, the gap in resources per capita ASSE / IAMCs fell from 3.5 times in 2005 to 1.3 in 2012 [56]. Nevertheless, differences remain for those not covered via FONASA-Uruguay, as ASSE does not receive per capita for these people, but a total amount of resources from general revenues that, since the start of the reform, have been increased to reach greater equalisation [49].

The reform also established a maximum threshold for contributions, above which FONASA-Uruguay returns the surplus [60], and included for-profit private insurance as an option for directing payroll contributions. Therefore, despite the new financing arrangements addressing redistribution, by eliminating proportionality at the highest incomes it circumscribes to some sectors of the society. In turn, it implies less availability of resources to improve the quality of services both at the ASSE and the IAMCs and broadens possibilities for the upper-middle and upper classes to opt-out.

In sum, Chile and Uruguay continue to rely on payroll contributions as the primary eligibility criterion. Still, because they complement social insurance with (residual) subsidisation through the public schemes, both countries perform highly in coverage (first dimension of policy outputs) in terms of numbers but not equally well regarding the composition. Chile achieves some unification in public insurance by including people independently of their ability to contribute, but the policy architecture keeps a two-tier health system that segments the population between those who can afford private options (i.e. the middle and upper-middle classes) and those who cannot. Uruguay advanced unification by including all contributors to social security in a single fund (i.e. FONASA-Uruguay). However, the population is still segmented by the ability to contribute to social security, as the ones who lack FONASA-Uruguay can only be covered at ASSE (i.e. via subsidisation). Moreover, the new institutional conditions have enhanced the options of the better-off to taking out private insurance (i.e. segmentation by income level), although to a lesser degree than in Chile.

Regarding benefits, in Chile, the comprehensiveness of services in the public sector and the guarantees included in the AUGE-GES Plan have promoted higher equality throughout the entire population, although there are differences between people who opt for private insurance due to the individual negotiation of their health plans, which depend on the payment capacity [38]. Nevertheless, the comprehensiveness of benefits is counterbalanced by the design of service delivery that may reinforce segmentation at various levels. Although access to public services has to do with the enrolment in FONASA-Chile, either via payroll contributions or subsidies, private delivery relies on the payment capacity and, therefore, is restricted to people who can afford co-payments, either being holders of private insurance (i.e. upper and upper-middle classes) or covered by FONASA-Chile via contributions to social security (i.e. middle classes). The latter because the public insurance financially supports services with private suppliers (´Free Choice Modality`) to contributors, whereas the subsidised population can only seek services at the public network of providers (´Institutional Modality`) [67]. However, the AUGE-GES Plan has partially contributed to overcoming differences (i.e. for the set of health problems included), as the law compels the fulfilment of the guarantees of quality, financial protection, and timely attention, even if this implies the purchasing of private services, thus suppressing the differences between groups.

In Uruguay, all providers included in the SNIS have to guarantee the Comprehensive Benefit Plan (Plan Integral de Asistencia a la Salud, PIAS) [49, 55], which tackles inequities by guaranteeing the same comprehensive benefits to the entire population. Nonetheless, as in Chile, the model of delivery involves some barriers for equitable access to such benefits. IAMCs are the main provider of services, but only FONASA-Uruguay contributors (or people taking out voluntary insurance) can hold coverage at the IAMCs, which may hamper the accessibility and timely attention of the rest of the population, who have to seek services in a more restricted network of providers. The reform also regulated moderation fees for all services included in the PIAS, but premia and co-payments remain scarcely regulated at for-profit private insurance, as well as at the IAMCs for non-PIAS services and amenities offered on a voluntary basis, thus remaining unequally accessible [68].

Finally, the massive presence and high deregulation of market-based options in Chile [59] set institutional conditions that may seriously hamper the efforts towards universalism. It, along with the targeting of most subsidies at the poor, raise co-payment and other OOP spending, hindering financial protection. Although the country reduced OOP spending from 48.8% of the total health expenditure in 2000 [3] to 32% in 2015, it remains high compared with the 20% average of OECD countries [69]. Latest analyses indicate that households devote over 5% of their total spending to health services [70, 71] and between 1.9 and 3.6%^4^ of households experience catastrophic health expenditures [72]. In such a landscape, people relying on private insurance face 39% higher OOP spending vis-à-vis those covered by FONASA-Chile [73].

Purchasing of medicines and co-payments for health services are two central sources of OOP (see [70, 74]). Regarding co-payments, even if they are progressive at the public network of providers, FONASA-Chile holders still pay a percentage for some services, and have to afford unregulated co-payments when seeking services outside the public network (i.e. under the Free Choice Modality). In turn, for those insured at the ISAPREs co-payments are deregulated^5^ and depend on the specific plan purchased and the provider, so that people with cheaper plans at ISAPREs co-pay higher [38]. Besides, by focusing on the poor, state subsidies neglect the middle-income earners, who need to rely on their own economic resources.

Additionally, the constraints of quality and resources for service delivery in the public network, together with the deregulation of co-payments at private providers, hinder the high performance that the country exhibits in service utilisation,^6^ raising inequities both within the public sector and between the public and private sectors. Studies show that within FONASA-Chile, contributors display higher levels of service utilisation vis-à-vis people subsidised, especially in specialist treatments [75]. In turn, FONASA-Chile holders have less service coverage of non-communicable diseases than people at the ISAPREs [38, 72]. Thus, there is a positive correlation between service utilisation, on the one hand, and higher income, and affiliation at ISAPREs, on the other [38, 76]. Nevertheless, such a correlation also indicates that the higher utilisation of services relies on the households´ investments rather than on more financial protection at the ISAPREs.

Last but not least, the introduction of the AUGE-GES Plan has not gone hand in hand with a coherent strengthening of the resolutive capacity of the public network to deliver quality services within the pre-established deadlines. Consequently, a significant amount of public resources are currently conducted to purchase private services [77] to meet the guarantees of service timing [38], constraining the availability of resources to improve the public delivery of services and, therefore, the quality of benefits acceded by lower-income groups vis-à-vis the ones with the ability to pay.

Unlike the ISAPREs in Chile, IAMCs historically contributed to meeting the health care needs of the population in Uruguay despite not being public, although there were relevant disparities founded on people’s participation in the labour market [48]. The health reform recognised this legacy and kept the IAMCs in a central role within the SNIS, as well as strengthened the regulations (e.g. IAMCs cannot reject the coverage of any FONASA-Uruguay holder, moderation fees and services are defined by the MSP) in order to ensure more equitable access to health care [68]. Besides, the state continues to subsidise the poor by providing free-services cards at ASSE [49]. These measures expressed in the reduction of the OOP spending from 20% in 2000 to 16% in 2014 being one of the lowest in the region [78].

Nevertheless, households still have to deal with moderation fees at the IAMCs that are not adjusted by payment capacity [56, 79] and differ across providers [60]. Consequently, the current structure of payment still involves income-related gaps in the opportunity to access health care. Indeed, the analysis of the financial performance of the health reform indicates that moderation fees primarily moderate the demand for services of low-income earners [68, 80]. Still, the utilisation of services does not present notorious differences between people at ASSE and the IAMCs. Overall, the rates of utilisation in the case of health need are about 97% [56].

The reform also included market-based options (i.e. for-profit insurance) into the SNIS and, in doing so, allowed people to make a proportion of their payroll contributions to these institutions. This risks facilitating the opting-out of the wealthier population, but also allows for the retention of their contributions within the system. Besides, the regulation of these institutions is still weak (e.g. they can determine co-payments and cream the population).

To sum up, the endurance of fragmentation in both health systems translates into differences in the sociodemographic profiles of the population covered. This, although to a lesser extent in Uruguay, implies the reproduction of inequalities created in other social domains. For instance, workers with lower wages, who are frequently women, are underrepresented both at the ISAPREs in Chile and at the IAMCs and for-profit insurance in Uruguay [75]. This is further reinforced by the possibility of ISAPREs in Chile and for-profit private insurance in Uruguay of creaming the population, thus discriminating against those with higher health needs (e.g. the older and women) through the requirement of higher premia for taking out the health plans. Therefore, the health system replicates the gender and labour market inequalities.

## Conclusion

The study shows significant differences in the process of reforms for UHC undertaken by Chile and Uruguay. It also points out the relevance of the different policy instruments that make up the policy architectures to reverse, or conversely deepen, segmentation across groups of the population.

In Chile, the introduction of the AUGE-GES Plan has addressed quality and equity for a set of health problems. Still, the process of reform has neither involved structural changes to the health system nor guaranteed quality access and financial protection for all. Rather, the subsidiary role assigned to the state defined by the reforms that in the early 1980s built the dual public-private health system persists up to date. Moreover, the subsidiary performance of the state is not just because of targeting subsidies at the poor, but also because it indirectly subsidises the private options (e.g. by allowing the ISAPRES to cream the population despite receiving social security funds, or by purchasing services from private providers rather than strengthening the capacity of the public network). The country certainly has not only advanced formal coverage but also the delivery of increasingly comprehensive services, all of which contributes to UHC. Other instruments of policy architecture, namely the financing mechanisms, the lack of regulation of market-based options, and the arrangements for service delivery, however, set institutional conditions that support the segmentation of the population by their ability to pay. In doing so, these policy instruments hinder the quality of services in the public sector, increase health-related financial risks faced by the whole population, but in particular by the middle classes when seeking private options, and hamper equity in accessing health services. As it happens in other southern countries see [3, 5], the analysis presented shows that the prominent participation of the market does not just bring segmentation but also erodes the performance of the public sector and the unification of the other components of the policy architecture.

Following a different approach and, perhaps more importantly, rooted in a substantially different starting point, the 2007 health reform in Uruguay advanced access to health care, enhancing generous services and financial protection more than ever before. The reform significantly tackled the fragmentation that featured in the system since its foundation. Nevertheless, policy outputs reveal that the novel policy architecture has not completely suppressed segmentation between population covered via social security and subsidisation. Still, there have been remarkable advances, especially regarding the generosity of benefits, the strengthening of quality at the public sector, and the higher solidarity and progressive allocation of resources. Although, by incorporating market-based insurance as recipients of social security revenues, the new policy architecture might also open up new possibilities for segmentation.

However, the analysis presented unveils shortcomings in the unification of the policy architecture in Uruguay, displaying more evident advances and structural transformations in some policy instruments (e.g. financing arrangements and benefits) meanwhile others (e.g. service delivery) have had slighter adaptations.

In sum, both countries exhibit very high population coverage, which is a condition for UHC, but equity gaps in access to quality services and financial protection remain. Then, the current situation in these two countries may be described as a demanding transition on the path towards universalism in health care. The welfare literature stresses the relevance of middle classes to reach universal social policies and middle classes are bigger than ever before in LA [81] which raises their possibility of influencing policy decision making. In such a scenario, overcoming differences in quality and timely attention across the types of insurance and providers constitutes an essential condition to avoid further segmentation of the population and set the institutional conditions to promote the middle classes’ willingness to support further investments that allow quality health care and financial protection for all, independently of payment capacity and previous performance.

The analysis carried out demonstrates the yields of using an analytical framework that addresses the policy architecture to reach a more profound understanding of the institutional arrangements behind the policy outputs. Still, this cross-sectional picture may benefit from a historical lens that addresses the trajectory of the health policy over time and its relationships with other elements of the political economy in each country, thus illuminating the enablers and constraints for the institutional transformations required to continue the advances towards UHC.

There are many other areas beyond the scope of this study that need further research and may benefit from drawing upon a social policy approach. Just to mention some and with no intention of being exhaustive, it is fundamental to provide a more accurate account of the institutional constraints of the health care systems to boost more equitable health care from gender and life-course perspectives. Likewise, the design of policy strategies to tackle segmentation may be gained from a better understanding of the health system anchors in the broader political economy (e.g. how the policy of health care deals with the aims and rationale of the wider social policy regime in each country, such as the role assigned to the state or the levels of commodification for accessing social welfare). Also, it may be helpful to grasp how the dynamics of the state-society relationship contribute to reproduce, or transform, the current policy outputs (e.g. how the policy instruments promote some practices for accessing health care and specific patterns of relationship with the public and private sectors in some groups of the population).

## Supplementary information

**Additional file 1 Table 1.** Contemporary policy architecture of health care in Chile and Uruguay. **Table 2.** Policy outputs of health care, Chile and Uruguay.

## Data Availability

Not applicable.

## Abbreviations

ASSE: Administración de los Servicios de Salud del Estado de Uruguay
AUGE-GES: Plan de Acceso Universal a Garantías Explícitas en Salud
BA: Basic Universalism
EU: Efficient Universalism
FONASA-Chile: Fondo Nacional de Salud de Chile
FONASA-Uruguay: Fondo Nacional de Salud de Uruguay
IAMCs: Instituciones de Asistencia Médica Colectiva de Uruguay
ISAPREs: Instituciones de Salud Previsional de Chile
LA: Latin America
OOP: Out of Pocket spending
PIAS: Plan Integral de Asistencia a la Salud
PHC: Primary Health Care
SNIS: Sistema Nacional Integrado de Salud de Uruguay
SPF: Social Protection Floor
UHC: Universal Health Coverage
UN: United Nations

## Footnotes

1 Yet, armed forces, the police, and a few categories of employees (e.g. municipal employees) have kept separated schemes with their own funds of resources.

2 FONASA-Uruguay corresponds to a single fund that pools all payroll contributions, whereas FONASA-Chile is the public insurance that has its own fund in which it pools revenues only from people covered by this insurance.

3 Levels of welfare were determined by a cluster analysis that included indicators on housing, comfort, education, and employment.

4 Calculations range depending on the threshold used to define catastrophic health expenditure, that is, the household’s spending on health care exceeds 40% or 30% of their remaining income after meeting subsistence needs.

5 Co-payments for AUGE-GES services are regulated

6 Data from the CASEN survey 2017 indicates that less than 1% of people experiencing any need for health care in the last 3 months reported not reaching the service (involuntarily).
