# Supplementary material for: Tackling segmentation to advance universal health coverage: analysis of policy architectures of health care in Chile and Uruguay
Source: Int J Equity Health. 2020 Jul 31;19:106. doi: 10.1186/s12939-020-01176-6 (PMC7362498; doi:10.1186/s12939-020-01176-6)
Supplement: Supplementary file 1 — Additional file 1 Table 1. Contemporary policy architecture of health care in Chile and Uruguay. Table 2. Policy outputs of health care, Chile and Uruguay. [file 12939_2020_1176_MOESM1_ESM.docx]

**Table 1: Contemporary policy architecture of health care in Chile and Uruguay**

| **Policy instruments** | **Chile** | **Uruguay** |
| --- | --- | --- |
| *Eligibility criteria* | - Payroll contributions: Formal workers^[[1]](#footnote-1)^ and family-dependents are eligible either for the public (FONASA-Chile) or private (ISAPREs) insurance. Nonetheless, ISAPREs can require the payment of extra premia. - Need: Vulnerable groups and the poor are eligible for subsidised enrolment in FONASA-Chile. | - Payroll contributions: Formal workers, pensioners, and family-dependents are eligible for the social security scheme, which allows coverage by any provider of the SNIS (i.e. ASSE, IAMCs or for-profit Private insurance)^[[2]](#footnote-2)^. Nonetheless, for-profit Private insurance can require the payment of extra premia - Citizenship: All citizens not covered through social security are eligible at the public scheme and covered through the public provider (ASSE). |
| *Funding* | - Revenue sources - Payroll taxes (monopartite - only workers) directed either at the public or private insurance (´Opt-out`) - General revenues - Voluntary premia to ISAPREs (i.e. complementary) - Co-payments: FONASA-Chile does not charge co-payments for PHC and for secondary/tertiary services co-payments are progressive. ISAPREs charge unregulated co-payments. - Pooling of resources and solidarity - FONASA-Chile: single fund (general revenues + contributions) – solidarity in resource allocation. - ISAPREs: multiple funds – no solidarity (individual insurance).   Contributions not pooled into a single fund (directing contributions to FONASA-Chile or ISAPREs) limits redistribution. | - Revenue sources - Payroll taxes (tripartite) directed to the single fund (FONASA-Uruguay) - General revenues - Voluntary premia to Private insurance - Co-payments: ASSE does not charge co-payments. IAMCs charge moderation fees determined by the MSP for some services. For-profit private insurance charge unregulated co-payments. - Pooling of resources and solidarity - Social security: single fund (FONASA-Uruguay) with all contributions – progressive revenue collection, and solidarity in resource allocation.   Exclusion of non-contributors limits redistribution. |
| *Benefits* | - Definition of benefits: Ministry of Health. - Benefits included - FONASA: All services - ISAPREs: Minimum package + other benefits/services according to the specific plan purchased. | - Definition of benefits: Ministry of Health. - Benefits included: All services (PIAS). |
| *Service delivery* | - Fragmented - Public providers: mainly population enrolled in FONASA-Chile (poor and lower middle class). - For-profit private providers: enrolled in ISAPREs (upper and upper-middle classes) and enrolled at FONASA-Chile via contributions (middle class by ´Free Choice Modality`) + Services included in the AUGE-GES Plan purchased by FONASA. | - Moderately fragmented - Public provider: mainly population not contributing to social security (poor). - IAMCs (private not-for-profit): population of the social security scheme (middle classes). - Private (for-profit): population of the social security scheme and holders of private insurance (upper and upper-middle classes). |
| *Outside options* | A large number of unregulated market-based options (insurance and providers).  Outside market-based options integrated into the system. | A large number of regulated not-for-profit providers (IAMCs).  A small number of moderately regulated market-based options (insurance and providers).  Outside option integrated into the system. |

Source: Own elaboration based on BPS (2019) (1), Cid *et al.* (2014) (2), Frenz *et al.* (2018) (3) and Oreggioni (2015) (4)

**Table 2: Policy outputs of health care, Chile and Uruguay**

|  | **Chile** | **Uruguay** |
| --- | --- | --- |
| *Coverage* | Massive coverage: 95.2% of the population in 2017 ^i^  Segmentation of the population ^i^:   - FONASA: 78% of the population   Poor, lower-middle and middle classes: 92% of people in the 1^st^ income-decile vs 25% in the 10^th^ income-decile.  Higher health risks: 80.6% of females vs 75.2% of males; about 85% of 60 or more years vs. near 74% of the working-age population.   - ISAPREs: 14.4% of the population   Upper-middle and middle classes: 2% of people in the 1st income-decile vs 68.2% in the 10^th^ income-decile.  Lower health risks: 13.1% of females vs 15.7% of males; about 8% of 60 or more years vs. near 16% of the working-age population.   - Other (e.g. Army forces, Police): 2.8% | Massive coverage: 98.4% of the population in 2014 ^ii^ ^[[3]](#footnote-3)^  Segmentation of the population:   - ASSE: 30.3% ^iii^   Poor, lower-middle: 42.3% of people with potential vulnerabilities in welfare vs 3.2% of people with high welfare (urban areas) ^iv^.   - IAMCs: 63% of the population ^iii^   Middle and upper-middle classes: 47.4% of people with potential vulnerabilities in welfare vs 81.5% of people with high welfare (urban areas) ^iv^.   - Private for-profit: 2.3% ^iii^   Upper class: 10.3% of people with high welfare vs. 0.6% of people with potential vulnerabilities in welfare (urban areas) ^iv^.   - Other (e.g. Army forces, Police): 4.9% ^iv^ . |
| *Generosity* | Allocation of resources per capita by calculation of health risks (progressive) within the public sector (FONASA and public providers). Less availability of resources per capita at the public sector vis-à-vis private options ^v^. | Allocation of resources per capita by calculation of health risks (progressive). Still, slightly fewer resources per capita allocated to the public sector (ASSE) ^iii^ ^vi^. |
| *Financial protection* | State subsidies for the poor and vulnerable population (enrolment in FONASA and services with public providers), for everybody holding public insurance in health problems included at the AUGE-GES Plan, and for all against catastrophic diseases.  Overall OOP spending reached 32% in 2015 ^vii^. It is lower within households enrolled at FONASA vis-à-vis those holding private insurance ^viii^.  Overall, service utilisation is high. Unmet health needs increase as income level decreases and are higher at FONASA vis-à-vis holders of private insurance. | State subsidies for the poor (enrolment and services at the ASSE) and for all against catastrophic diseases.  Overall OOP spending reached 16% in 2014 ^vii^. It is lower within households at ASSE vis-à-vis those at IAMCs or private insurance.  Overall, service utilisation is high. Unmet health care needs are similar at ASSE and the IAMCs ^vi^. |

Source: Own elaboration based on

^i^  Observatorio Social CASEN (2018) (5)

^ii^ Martínez (2015) (6)

^iii^ Ballart and Fuentes (2018) (7)

^iv^  Midaglia et al (2012) (8)

^v^ Frenz et al (2018) (3)

^vi^ Oreggioni (2015) (4)

^vii^  WHO (2019a) (9)

^viii^ Minsal-Chile (2015) (10)

1. BPS. Afiliación mutual: Banco de Previsión Social (BPS); 2019 [Available from: <https://www.bps.gub.uy/9516/afiliacion-mutual.html>.

2. Cid C, Aguilera X, Arteaga O, Barría S, Barría P, Castillo C, et al. Informe final Comisión Asesora Presidencial para el estudio y Propuesta de un Nuevo Régimen Jurídico para el Sistema de Salud Privado. Santiago: Comisión Asesora Presidencial para el Estudio y Propuesta de un Nuevo Régimen Jurídico para el Sistema de Salud Privado; 2014.

3. Frenz P, Siches I, Aguilera X, Arteaga O, Cid C, Estay R, et al. Propuesta para una reforma integral al financiamiento de la salud en Chile. Santiago-Chile: Comisión ESP-COLMED, Escuela de Salud Pública Universidad de Chile y Colegio Médico de Chile A.G.; 2018.

4. Oreggioni I. El camino hacia la cobertura universal en Uruguay: Cobertura poblacional del Sistema Nacional Integrado de Salud. In: Carrasco P, Fernández Galeano M, Fuentes G, Levcovitz E, Olesker D, Oreggioni I, editors. Economía, política y economía política para el acceso y la cobertura universal de salud en Uruguay. Montevideo-Uruguay: Organización Panamericana de la Salud (OPS); 2015. p. 47-79.

5. Observatorio Social CASEN. Salud. Síntesis de resultados. Santiago, Chile: Ministerio de Desarrollo Social; 2018.

6. Martínez B. Salud. In: Dutto M, editor. Reporte Uruguay 2015. Montevideo-Uruguay: Ministerio de Desarrollo Social y Oficina de Planeamiento y Presupuesto; 2015. p. 73-89.

7. Ballart X, Fuentes G. Gaining public control on health policy: the politics of scaling up to universal health coverage in Uruguay. Social Theory and Health. 2018;17(3):348-66.

8. Midaglia C, Antía F, Castillo M, Sanguinetti M. Distribución de vulnerabilidades y estrategias públicas de protección social. Montevideo-Uruguay: Departamento de Ciencia Política, Facultad de Ciencias Sociales Universidad de la República 2012.

9. WHO. Global Health Expenditure Database. In: (WHO) WHO, editor. 2019.

10. MINSAL-Chile. Informe final: Gasto catastrófico y de bolsillo en salud para el período 1997 – 2012. Santiago-Chile: Ministerio de Salud de Chile; 2015.

1. Contributions to social security are mandatory for salaried workers and, from 2019, self-employed workers earning over five minimum monthly wages (Law 21.133). [↑](#footnote-ref-1)
2. With the current regulation ASSE and IAMCs cannot reject individuals or provide partial coverage (i.e. by excluding some services) (Huber and Stephens, 2012). Nonetheless, it does not apply to private insurance, meaning that in practice contributions is not a sufficient criterion to hold private insurance. [↑](#footnote-ref-2)
3. Although formally everyone is covered, this figure is based on self-reports. [↑](#footnote-ref-3)
